# Atlanta College of Physicians and Surgeons

**Published:** 1904-04

**Authors:** 


### ATLANTA COLLEGE OF PHYSICIANS AND SURGEONS.

THE Commencement Exercises of the Atlanta College of Physicians and Surgeons were held at the Grand Opera House, March 29, 1904. The degree of doctor of medicine was conferred upon the following: H. C. Allyn, R. E. Bowman, L. J. Burch, F. P. Calhoun, G. A. Dame, J. M. Dennis, C. C. Fargason, A. G. Fort, J. D. Griffin, F. C. Herring, W. E. Lipscomb, T. Lowry, R. S. Lucius, J. T. McCall, D. B. McMullen, S. B. Malone, J. D. Manget, J. W. Martin, W. D. Martin, H. L. Montgomery, L. T. Pattillo, O. E. Shankle, P. S. Smith, H. Stapleton, E. E. Strickland, J. B. Tanner, J. P. Turk, G. E. Weems, S. P. Wells, E. L. Williams and D. J. R. Youngblood. The honor graduates were: Ferdinand Phinizy Calhoun, J. D. Manget, P. S. Smith, O. E. Shankle and C. C. Fargason. Dr. W. S. Kendrick made a report for the year. He said, in part:

"In attendance upon the three departments of medicine, pharmacy and dentistry, there have been this year four hundred and thirty-nine students. Two hundred and fifteen of these have been in the medical department and thirty-one ask at your hands the degree of doctor of medicine; one hundred and ten have attended the college of pharmacy, and forty-four are candidates for the degree of graduate in pharmacy; one hundred and fourteen have been enrolled in the dental department, and of these a number will, at a future date, ask for the degree of doctor of dental surgery.

"The two hundred and fifteen medical students represent an increase of seven and one-half per cent., as compared with last year; one hundred and fifty-eight are from Georgia, twenty-one from Alabama, fourteen from Florida, seven from South Carolina, five from Texas, two from Mississippi, two from Tennessee, one from New York, one from Kentucky, one from Pennsylvania, one from Indian Territory, one from Oklahoma and one from Brazil."

